# SARS-CoV-2 Uses Nonstructural Protein 16 To Evade Restriction by IFIT1 and IFIT3

**DOI:** 10.1128/jvi.01532-22

**Published:** 2023-02-01

**Authors:** Craig Schindewolf, Kumari Lokugamage, Michelle N. Vu, Bryan A. Johnson, Dionna Scharton, Jessica A. Plante, Birte Kalveram, Patricia A. Crocquet-Valdes, Stephanea Sotcheff, Elizabeth Jaworski, Rojelio E. Alvarado, Kari Debbink, Matthew D. Daugherty, Scott C. Weaver, Andrew L. Routh, David H. Walker, Kenneth S. Plante, Vineet D. Menachery

**Affiliations:** a Department of Microbiology and Immunology, University of Texas Medical Branch, Galveston, Texas, USA; b Institute for Human Infections and Immunity, University of Texas Medical Branch, Galveston, Texas, USA; c World Reference Center for Emerging Viruses and Arboviruses, University of Texas Medical Branch, Galveston, Texas, USA; d Department of Pathology, University of Texas Medical Branch, Galveston, Texas, USA; e Department of Biochemistry and Molecular Biology, University of Texas Medical Branch, Galveston, Texas, USA; f Institute for Translational Sciences, University of Texas Medical Branch, Galveston, Texas, USA; g Department of Microbiology and Immunology, Johns Hopkins University, Baltimore, Maryland, USA; h Department of Molecular Biology, University of California, San Diego, California, USA; i Center for Biodefense and Emerging Infectious Disease, University of Texas Medical Branch, Galveston, Texas, USA; Loyola University Chicago

**Keywords:** 2'-O-methyltransferase, IFIT1, IFIT3, interferon-stimulated gene, NSP16, SARS-CoV-2, antiviral agents, coronavirus

## Abstract

Understanding the molecular basis of innate immune evasion by severe acute respiratory syndrome coronavirus 2 (SARS-CoV-2) is an important consideration for designing the next wave of therapeutics. Here, we investigate the role of the nonstructural protein 16 (NSP16) of SARS-CoV-2 in infection and pathogenesis. NSP16, a ribonucleoside 2′-*O*-methyltransferase (MTase), catalyzes the transfer of a methyl group to mRNA as part of the capping process. Based on observations with other CoVs, we hypothesized that NSP16 2′-*O*-MTase function protects SARS-CoV-2 from cap-sensing host restriction. Therefore, we engineered SARS-CoV-2 with a mutation that disrupts a conserved residue in the active site of NSP16. We subsequently show that this mutant is attenuated both *in vitro* and *in vivo*, using a hamster model of SARS-CoV-2 infection. Mechanistically, we confirm that the NSP16 mutant is more sensitive than wild-type SARS-CoV-2 to type I interferon (IFN-I) *in vitro*. Furthermore, silencing IFIT1 or IFIT3, IFN-stimulated genes that sense a lack of 2′-*O*-methylation, partially restores fitness to the NSP16 mutant. Finally, we demonstrate that sinefungin, an MTase inhibitor that binds the catalytic site of NSP16, sensitizes wild-type SARS-CoV-2 to IFN-I treatment and attenuates viral replication. Overall, our findings highlight the importance of SARS-CoV-2 NSP16 in evading host innate immunity and suggest a target for future antiviral therapies.

**IMPORTANCE** Similar to other coronaviruses, disruption of severe acute respiratory syndrome coronavirus 2 (SARS-CoV-2) NSP16 function attenuates viral replication in a type I interferon-dependent manner. *In vivo*, our results show reduced disease and viral replication at late times in the hamster lung, but an earlier titer deficit for the NSP16 mutant (dNSP16) in the upper airway. In addition, our results confirm a role for IFIT1 but also demonstrate the necessity of IFIT3 in mediating dNSP16 attenuation. Finally, we show that targeting NSP16 activity with a 2′-*O*-methyltransferase inhibitor in combination with type I interferon offers a novel avenue for antiviral development.

## INTRODUCTION

Since its emergence late in 2019, severe acute respiratory syndrome coronavirus 2 (SARS-CoV-2) has caused major damage to the global populace through mortality (1), morbidity (2), and social and economic disruption (3). While the pandemic may be seen as shifting to endemicity, the continued threat of epidemic waves remains due to waning immunity and/or the emergence of new SARS-CoV-2 variants of concern (4). Moreover, future outbreaks caused by CoVs seem possible considering previous epidemics this century caused by SARS-CoV and Middle East respiratory syndrome (MERS)-CoV (5). Therefore, there is a need to expand our understanding of SARS-CoV-2 and identify additional avenues for treatment.

CoVs encode an array of viral effectors that subvert host immunity to allow for successful replication (6, 7). However, variations in function and effect across the CoV family indicate a need to functionally test these effectors in viral replication and pathogenesis studies. CoV nonstructural protein (NSP16), a ribonucleoside 2′-*O*-methyltransferase (MTase), catalyzes the transfer of a methyl group to the viral RNA cap structure (8, 9). This modification to the viral RNA cap is thought to prevent recognition by the host RNA sensor MDA5 and effectors in the interferon-induced protein with tetratricopeptide repeats (IFIT) family (10, 11). Reliance on 2′-*O*-methylation has been observed in a broad range of virus families that either encode their own 2′-*O*-MTases (12), rely on a host 2′-*O*-MTase (13), or simply “snatch” host mRNA caps to incorporate into their own viral RNA (14). Disrupting the ability of these viruses to mimic host RNA cap structure results in a range of attenuation phenotypes (10, 13, 15, 16).

In this work, we confirmed the importance of SARS-CoV-2 NSP16 to viral infection and pathogenesis. Building from previous studies on CoV 2′-*O*-MTases, we disrupted via mutagenesis a conserved lysine-aspartic acid-lysine-glutamate (KDKE) catalytic tetrad necessary for NSP16 MTase function (11, 17). We found that the NSP16 MTase mutant (dNSP16) was attenuated *in vitro* in the context of type I interferon (IFN-I) activity. Additionally, we observed reduced disease and viral loads for dNSP16 in the hamster model. Importantly, we showed that the IFN-stimulated genes (ISGs) IFIT1 and IFIT3 mediate dNSP16 attenuation. Finally, targeting NSP16 activity with the MTase inhibitor sinefungin increased the sensitivity of wild-type (WT) SARS-CoV-2 to IFN-I treatment. Together, these findings demonstrate a key role for NSP16 in SARS-CoV-2 immune evasion and potentially identify CoV 2′-*O*-MTase function as a target for novel therapeutic approaches (18).

## RESULTS

### dNSP16 has no replication defect.

**FIG 1:**
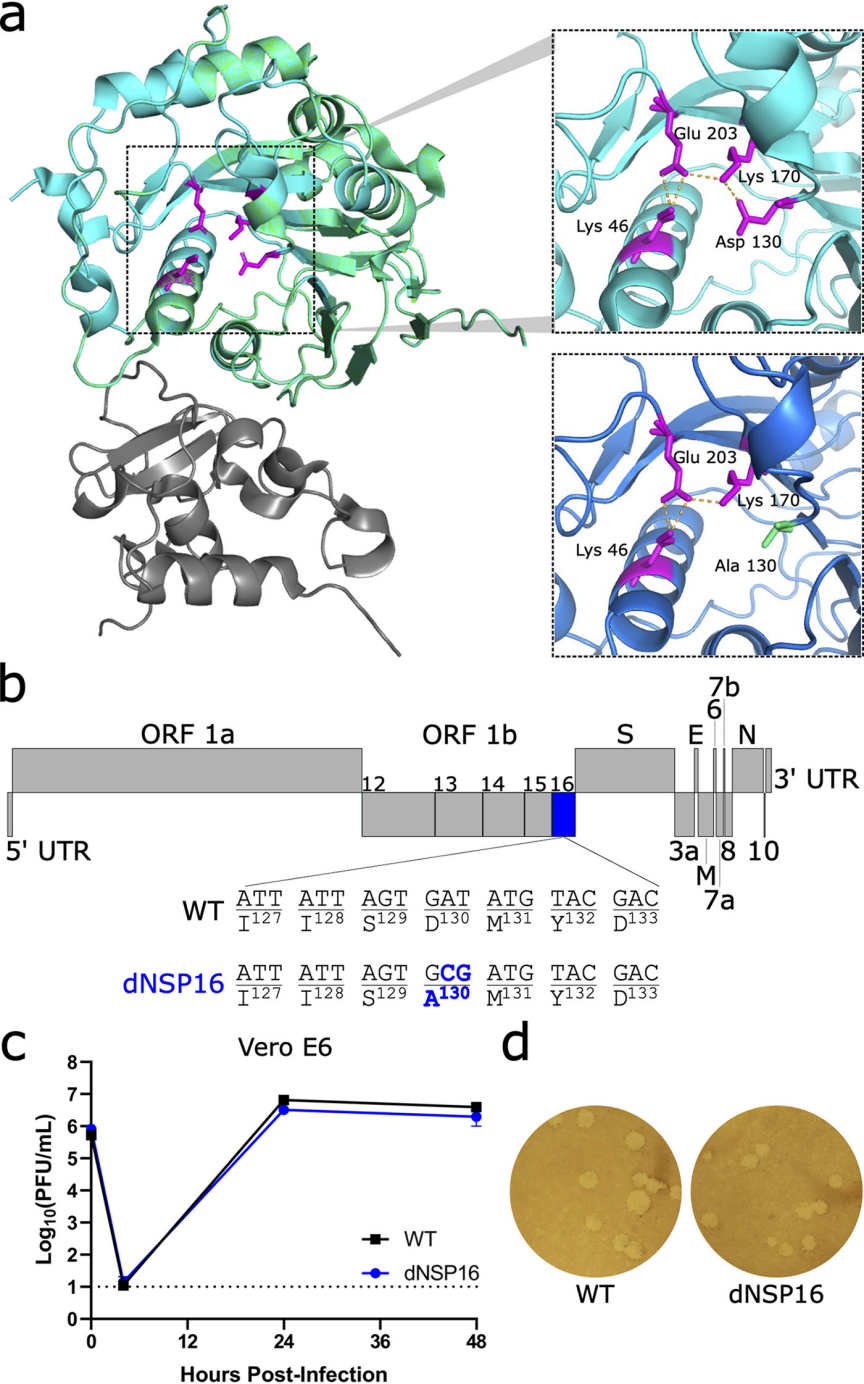
dNSP16 has no replication defect. (a) Severe acute respiratory syndrome coronavirus 2 (SARS-CoV-2) NSP16 (green) in complex with scaffold NSP10 (gray). The upper inset shows the KDKE catalytic tetrad (in magenta, with amino acids labeled) with polar contacts shown by orange dashed lines. The lower inset shows mutation of the KDKE motif to KAKE (D130A). The structural modeling demonstrates a loss of a hydrogen bond between K170 and A130. Structures based on Protein Data Bank ID 6W4H with homology model made using Swiss-Model (18). (b) Schematic of the SARS-CoV-2 genome, drawn to scale, with NSP16 highlighted in blue and the engineered two-base change indicated, resulting in coding change D130A. (c) Replication of WT (black) and dNSP16 (blue) in Vero E6 cells, multiplicity of infection (MOI) = 0.01 (*n *= 3). Means are plotted with error bars denoting standard deviation. The dotted line represents the limit of detection. (d) Plaque morphology of WT and dNSP16 on Vero E6 cells. ORF, open reading frame; UTR, untranslated region; WT, wild-type.

To investigate the contribution of NSP16 to SARS-CoV-2, we constructed dNSP16 using our infectious clone of SARS-CoV-2 as previously described (19, 20). Briefly, we generated a 2-bp substitution, converting aspartic acid to alanine (D130A) in the conserved KDKE motif (Fig. 1a and b). This mutation is predicted to ablate MTase function; specifically, prior studies with purified SARS-CoV NSP16 have shown that the D130A mutation completely ablates MTase activity in a cell-free system (21). Prior CoV studies have confirmed the importance of this residue to CoV replication and pathogenesis (10, 11, 15, 22, 23). We also attempted to construct an NSP16 deletion virus by engineering an in-frame stop codon at the first amino acid position, but this deletion mutant failed to replicate. In IFN-deficient Vero E6 cells, dNSP16 displayed replication kinetics (Fig. 1c) and plaque sizes similar to those of WT (Fig. 1d). Together, these results suggest no significant impact on viral replicative capacity with the loss of NSP16 catalytic activity. Importantly, the D130A mutation was found to be stable in our rescued dNSP16 stock by Sanger sequencing, and we confirmed no common spike mutations in the region adjacent to the furin cleavage site that have been previously reported for virus stocks amplified on Vero E6 cells (24, 25) (Fig. S1).

### dNSP16 is attenuated in human respiratory cells.

**FIG 2:**
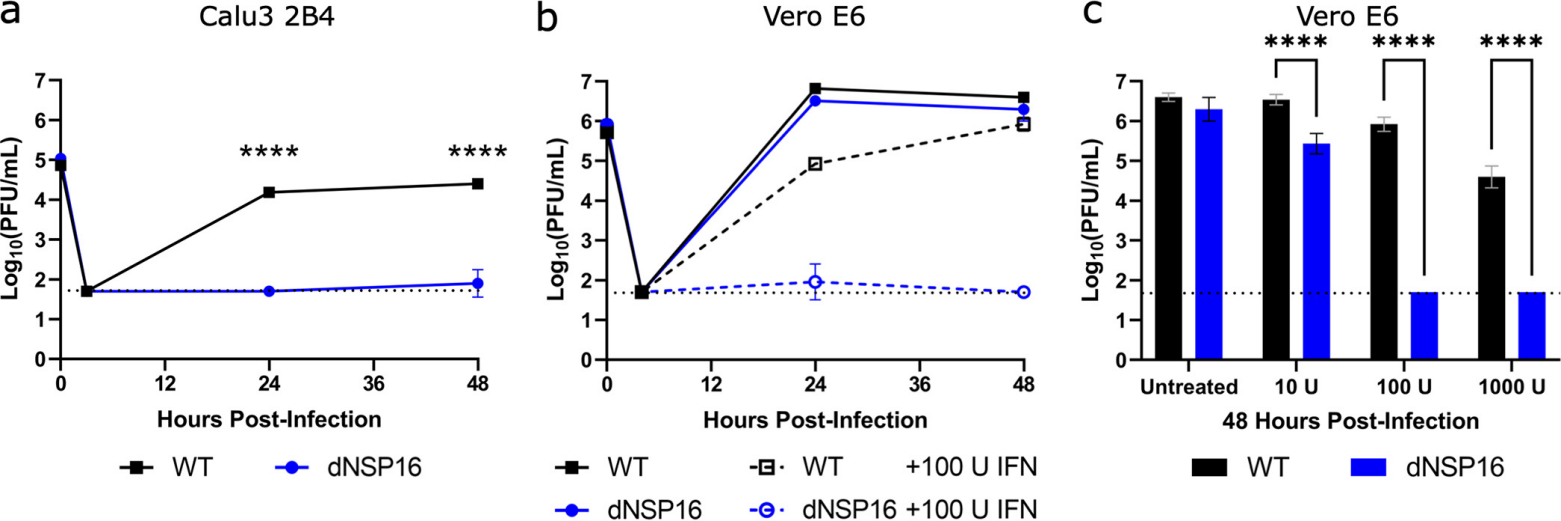
dNSP16 is attenuated in human respiratory cells and is more sensitive to type I interferon (IFN-I) pretreatment. (a) Replication of WT (black) and dNSP16 (blue) in Calu-3 2B4 cells, MOI = 0.01. ****, *P < *0.001: results of two-way analysis of variance (ANOVA) with Tukey’s multiple-comparison test (*α* = 0.05). (b) Replication of WT (black) and dNSP16 (blue) in Vero E6 cells without IFN-I (solid lines, data as in Fig. 1c), or with 100 U IFN-I pretreatment a day prior to infection (dashed lines); MOI = 0.01. (c) Comparison of the viral titers at 48 h postinfection from panel b, with additional treatment levels of IFN-I from the same experiment indicated. ****, *P < *0.001: results of two-way ANOVA with Tukey’s multiple-comparison test (*α* = 0.05). For all panels, the means are plotted with error bars denoting standard deviation (*n *= 3 for all data points). The dotted lines represent limits of detection.

While the dNSP16 mutant had no replicative attenuation in Vero E6 cells, phenotypes in these cells are often not representative of relevant cell types such as human respiratory cells (25–27). Therefore, we next evaluated dNSP16 in Calu-3 2B4 cells, a human lung carcinoma cell line. Compared to WT SARS-CoV-2, we observed significant attenuation of dNSP16 in Calu-3 2B4 cells (Fig. 2a). At both 24 and 48 h postinfection (HPI), WT SARS-CoV-2 displayed robust replication, whereas a 2.5 log_10_ decrease in replication was observed for dNSP16 at both time points. These results are consistent with similar findings for both SARS-CoV and MERS-CoV 2′-*O*-MTase mutants (15, 23). Together, the results confirm the requirement of NSP16 for successful SARS-CoV-2 infection of human respiratory cells.

### dNSP16 is more sensitive to type I IFN pretreatment.

A major distinction between Vero E6 and Calu-3 2B4 cells is their capacity to induce a type I interferon (IFN-I) response; while Calu-3 2B4 cells are IFN-I competent, Vero E6 cells do not induce IFN-I but do respond to it when treated exogenously. Therefore, we investigated the effects of IFN-I on the replication of dNSP16 relative to WT. Pretreating Vero E6 cells with 100 U of IFN-I, we noted a modest but significant decrease in WT infection compared to untreated cells (Fig. 2b). In contrast, Vero E6 cells pretreated with IFN-I resulted in 3.0 log_10_ and 4.2 log_10_ decreases in dNSP16 titer at 24 and 48 HPI, respectively. We observed a dose-dependent decrease in titer with respect to IFN-I pretreatment for both dNSP16 and WT; however, the effect on dNSP16 was more pronounced, especially at higher IFN-I concentrations (Fig. 2c). Overall, the results indicate that dNSP16 is more sensitive to IFN-I compared to WT SARS-CoV-2.

### dNSP16 is attenuated *in vivo*.

**FIG 3:**
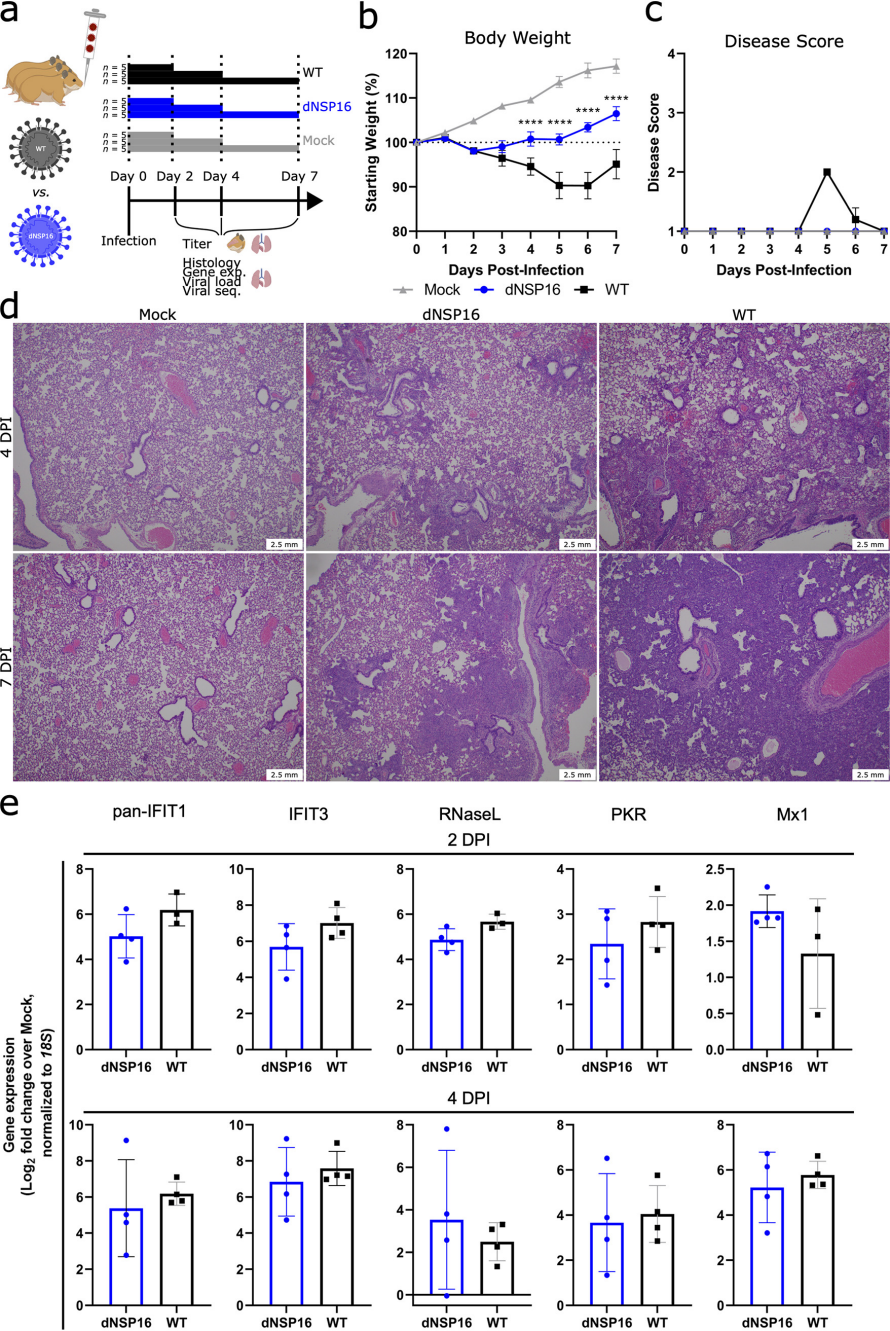
dNSP16 is attenuated *in vivo*. (a) Overview of experimental plan for hamster infections. A 100-μL inoculum of phosphate-buffered saline (mock) or 10^4^ plaque-forming units of either dNSP16 or WT was given intranasally to 4- to 5-week-old Syrian hamsters. At 2, 4, and 7 days postinfection (DPI), 5 animals from each infection group were sacrificed for organ collection. Some graphics were generated from BioRender.com. (b, c) Percent starting weights (b) and disease scores (c) for mock-, dNSP16-, or WT-infected hamsters. ****, *P < *0.001: results of a mixed-effects model (restricted maximum likelihood) with Tukey’s multiple-comparison test (*α* = 0.05); asterisks denote test results between WT- and dNSP16-infected hamsters at the indicated DPI. Means are plotted with error bars denoting standard error of the mean. (d) Hematoxylin and eosin staining of representative 5-μm-thick sections taken from left lung lobes. (e) Fold change (log_2_) of expression of the indicated immune genes from right middle lung lobes isolated from hamsters infected with the indicated virus, at either 2 or 4 DPI. For each panel, fold changes from dNSP16 or WT samples are measured relative to mock samples. Values from individual hamsters are plotted (symbols), as are means (bars). Error bars denote standard deviation. All samples were normalized to 18S expression, used as a reference.

We next asked whether the attenuation of dNSP16 we observed *in vitro* would manifest *in vivo*. We challenged Syrian (golden) hamsters, a model for SARS-CoV-2 infection studies (28), intranasally (i.n.) with 10^4^ PFU of dNSP16 or WT or with phosphate-buffered saline (PBS) as a mock-infection control (Fig. 3a). While both dNSP16- and WT-infected hamsters showed weight loss relative to the mock-infected control hamsters, the dNSP16-infected hamsters showed reduced weight loss compared to WT-infected hamsters (Fig. 3b). Moreover, the dNSP16-infected hamsters did not show signs of disease, and only the WT-infected hamsters displayed ruffled fur at 5 and 6 days postinfection (DPI) (Fig. 3c). Lung histopathologic findings were more severe for WT-infected hamsters compared to dNSP16-infected hamsters at both 4 and 7 DPI (Fig. 3d; Fig. S2). Both groups developed interstitial pneumonia, bronchiolitis, peribronchiolitis, perivasculitis, and perivascular edema. WT-infected hamsters experienced a greater degree of subendothelial edema and hemorrhage. Together, these results indicate that dNSP16 results in reduced disease in the hamster model of SARS-CoV-2 infection.

To explore why the disease phenotype differed in dNSP16-infected hamsters, we first evaluated changes in the host immune response following infection with dNSP16. Examining RNA from hamster lungs collected at both 2 and 4 DPI, we observed that both WT- and dNSP16-infected samples had increased gene expression in the small set of ISGs (IFIT1 paralogs, IFIT3, RNase L, PKR, and Mx1) (Fig. 3e) and other key immune genes (IFNγ, interleukin [IL]-1β, and IL-10) (Fig. S3) relative to mock-infected samples. However, no differences in lung expression were observed between WT- and dNSP16-infected hamsters. Our results suggest that loss of NSP16 activity may not drive differential expression of immune genes in the lungs but rather dNSP16 may be more sensitive to an immune gene(s) otherwise ineffective against WT SARS-CoV-2.

### dNSP16 replication is reduced *in vivo*.

**FIG 4:**
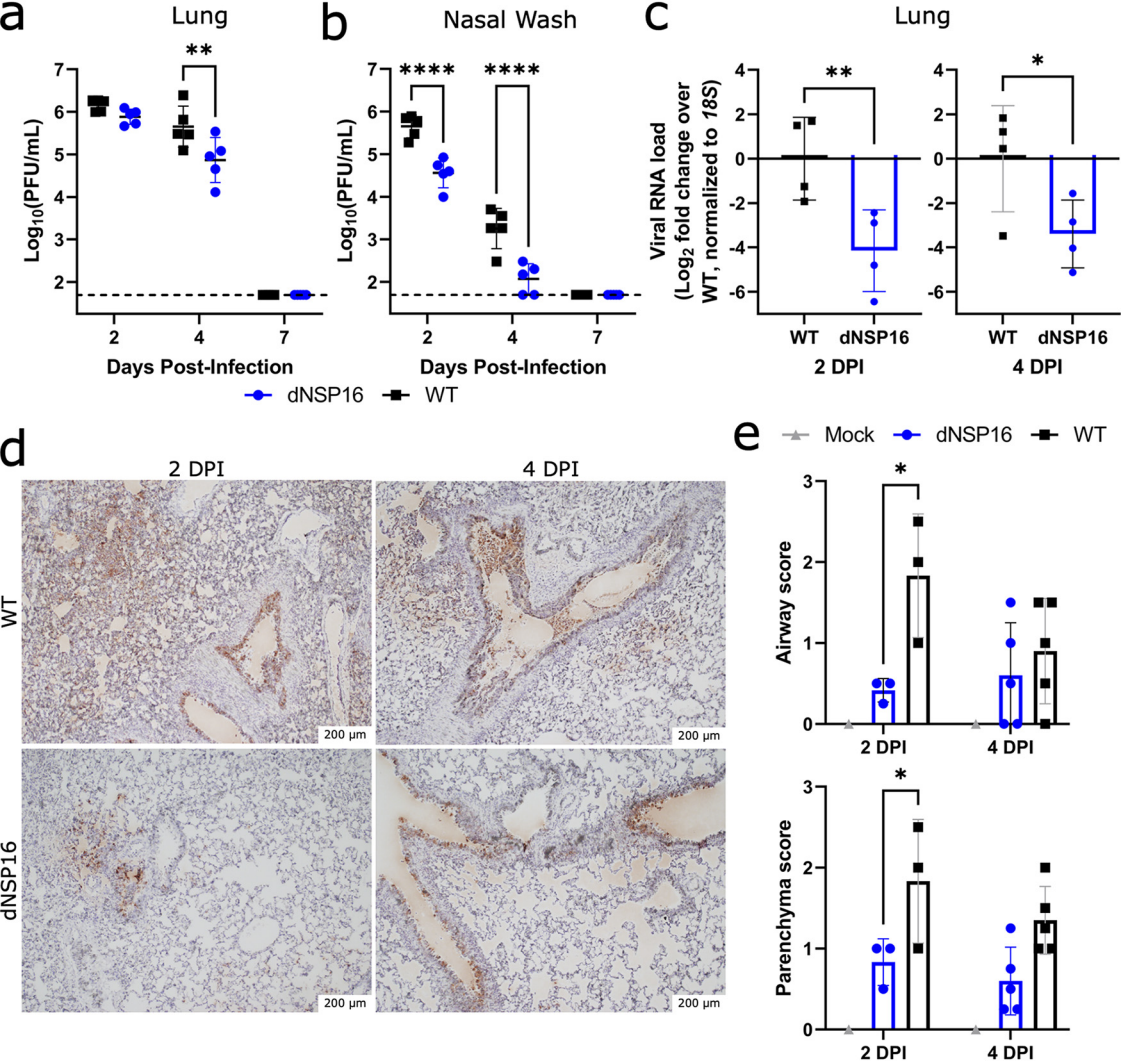
dNSP16 replication is reduced *in vivo*. (a, b) Comparison of viral titers from right cranial lung lobes (a) or nasal washes (b) from WT-infected (black) or dNSP16-infected (blue) hamsters sacrificed at the indicated day. **, *P < *0.01; ****, *P < *0.001: results of two-way ANOVA with Tukey’s multiple-comparison test (*α* = 0.05). Values from individual hamsters are plotted (symbols), as are means (black bars). Error bars denote standard deviation. The dotted lines represent the limits of detection. (c) Relative viral load detected in RNA extracted from right middle lung lobes isolated from hamsters infected with the indicated virus, at either 2 or 4 DPI. Fold change (log_2_) is given with respect to WT viral load. The values from individual hamsters are plotted (symbols), as are means (bars). Error bars denote standard deviation. All samples were normalized to 18S expression, used as a reference. *, *P < *0.05: results of a one-tailed unpaired *t* test. (d) SARS-CoV-2 nucleocapsid staining (brown) of representative 5-μm-thick sections taken from left lung lobes. (e) Scores of staining amounts for airway or lung parenchyma from samples stained as in panel d. *, *P < *0.05: results of a two-way ANOVA with Šídák's multiple-comparison test (*α* = 0.05).

We next evaluated viral load in dNSP16-infected versus WT-infected hamsters. Examining replication in the lung, we observed similar viral loads at 2 DPI between dNSP16- and WT-infected hamsters (Fig. 4a); however, by 4 DPI, dNSP16 titer was reduced. This delayed attenuation in the lung corresponds to previous reports for both SARS-CoV and MERS-CoV in mice (23, 29). However, nasal wash titers at both 2 and 4 DPI were lower for dNSP16-infected hamsters compared to WT-infected hamsters (Fig. 4b). These nasal wash titer data suggest that attenuation of dNSP16 occurs in the upper airway at an earlier time compared to lung and may demonstrate different tissue-mediated immune responses. Notably, while viral titers in the lung were equivalent at 2 DPI, SARS-CoV-2 RNA from the lung suggested reduced viral load for dNSP16-infected hamsters compared to WT-infected hamsters (Fig. 4c). This discrepancy may be attributed to differences in sensitivity between the two methods. Additionally, nucleocapsid-specific staining of lung tissue showed more pervasive viral presence in both the airways and the parenchyma for WT-infected tissues versus dNSP16-infected tissues (Fig. 4d and e; Fig. S4). Consistent with differences in fitness *in vivo*, targeted Sanger sequencing of viral RNA from the lungs at 4 DPI showed no signs of reversion in the dNSP16-infected hamsters (Fig. S5). Together, these results indicate that dNSP16 causes reduced disease and exhibits decreased viral replication *in vivo*, despite inducing a similar immune response to WT SARS-CoV-2 in the lung.

### Knockdown of IFIT genes partially reverses attenuation of dNSP16.

**FIG 5:**
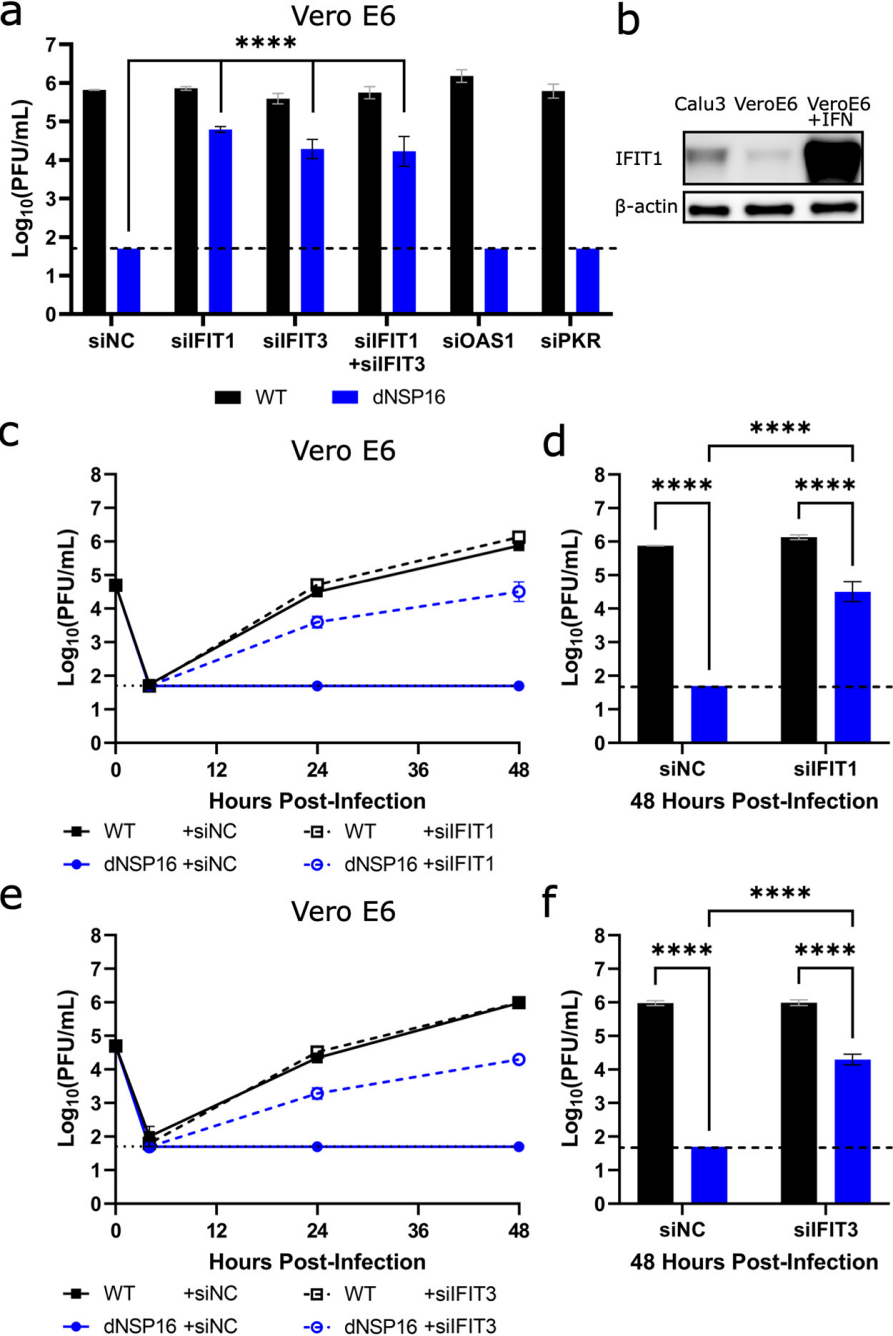
Knockdown of IFIT genes partially reverses attenuation of dNSP16. (a) Replication of WT (black) and dNSP16 (blue) in the context of small interfering RNA (siRNA) treatment. A total of 1.25 × 10^5^ Vero E6 cells/well were reverse transfected with 2 pmol total of the indicated siRNA construct(s) 2 days prior to infection and also pretreated with 100 U IFN-I 1 day prior to infection; multiplicity of infection (MOI) = 0.01. The data shown are from 48 h postinfection (HPI). Statistical comparisons on graph are with respect to siRNA control treatment (siNC). (b) Baseline IFIT1 protein expression in Calu-3 2B4 and Vero E6 cells, or Vero E6 cells 1 day poststimulation with IFN-I. (c) Viral replication kinetics for WT (black) or dNSP16 (blue) following treatment with anti-IFIT1 (dashed) or control siRNA (solid). A total of 1.25 × 10^5^ Vero E6 cells were reverse transfected with 1 pmol of the indicated siRNA construct 2 days prior to infection and also pretreated with 100 U IFN-I 1 day prior to infection; MOI = 0.01. (d) Comparison of the viral titers at 48 HPI from panel c; black = WT, blue = dNSP16. (e) Viral replication kinetics for WT (black) or dNSP16 (blue) following treatment with anti-IFIT3 (dashed) or control siRNA (solid). A total of 1.25 × 10^5^ Vero E6 cells/well were transfected with 1 pmol of the indicated siRNA construct 2 days prior to infection and also pretreated with 100 U IFN-I 1 day prior to infection; MOI = 0.01. (f) Comparison of the viral titers at 48 HPI from panel e; black = WT, blue = dNSP16. For panels a, d, and f, ****, *P < *0.001: results of two-way ANOVA with Tukey’s multiple-comparison test (*α* = 0.05). Means are plotted with error bars denoting standard deviation. For all panels, *n *= 3 biological replicates for all data points. The dotted lines represent the limits of detection.

Based on increased sensitivity to IFN-I, attenuation of dNSP16 is likely mediated by sensitivity to certain ISG effectors. Therefore, we focused on several ISGs known to target foreign nucleic acids, including the IFIT family (30), PKR (31), and OAS1 (32). We transfected Vero E6 cells with target or control small interfering RNA (siRNA), treated them with IFN-I, and then infected them with either WT SARS-CoV-2 or dNSP16. Whereas control siRNA treatment resulted in undetectable viral titers for dNSP16 at 48 HPI, consistent with the attenuating effect of IFN-I (Fig. 2b and c), we observed a significant restoration of viral titers with anti-IFIT1 siRNA treatment (Fig. 5a). Similarly, siRNA-induced knockdown of IFIT3, shown to stabilize IFIT1 and enhance its cap-binding function (33), resulted in a restoration of dNSP16 titers comparable to those observed with anti-IFIT1 siRNA. However, the combination of IFIT1 and IFIT3 knockdown had no additive impact in these studies. Notably, neither anti-PKR nor anti-OAS1 siRNA treatment significantly affected viral replication relative to control siRNA, despite confirming knockdown for all targets (Fig. S6). Together, the results suggest that both IFIT1 and IFIT3 play critical roles in the attenuation of dNSP16.

IFIT family members have previously been shown to recognize nonhost mRNA cap structures (34). Based on the initial siRNA screen (Fig. 5a), we next evaluated whether the differences in viral attenuation that we noted between dNSP16 and WT SARS-CoV-2 may be due to the presence of baseline IFIT1 expression in the cells we tested. We subsequently observed that Calu-3 2B4 cells expressed IFIT1 protein at baseline, whereas expression of IFIT1 in Vero E6 cells was low (Fig. 5b). However, upon stimulation of Vero E6 cells with IFN-I, we observed a robust induction of IFIT1 that may account for the dNSP16 attenuation we noted (Fig. 2c). We further examined the replication kinetics of dNSP16 in the context of IFIT1 knockdown (Fig. 5c). Whereas treatment with 100 U of IFN-I and control siRNA resulted in undetectable viral titers for dNSP16 at all time points tested, we observed partial restoration of viral titers for dNSP16 in the context of anti-IFIT1 siRNA treatment at both 24 and 48 HPI (Fig. 5c and d). While the role of IFIT1 has previously been noted for CoV 2′-*O*-MTases (15, 23), IFIT3 has only recently been shown to enhance IFIT1’s RNA-binding ability in human cells (33). Similar to IFIT1 knockdown, IFIT3 knockdown restored replication of dNSP16 at both 24 and 48 HPI (Fig. 5e and f). Since IFIT1 and IFIT3 share sequence homology, we also confirmed that both our anti-IFIT1 and anti-IFIT3 siRNA constructs were specific to their respective targets (Fig. S7). Coupled with the fact that combined anti-IFIT1/anti-IFIT3 siRNA treatment had no additive effect (Fig. 5a), these results suggest that both human IFIT1 and IFIT3 are necessary for attenuation of SARS-CoV-2 dNSP16.

Having shown that IFIT1 and IFIT3 were both necessary for attenuation of dNSP16, we next examined whether IFIT1 and/or IFIT3 were sufficient for attenuation of dNSP16 by transient transfection. To avoid species incompatibility and increase transfection efficiency, the hepatoma cell line Huh7 was used and showed a modest, but significant, 0.5 log_10_ reduction in dNSP16 replication compared to WT in Huh7 cells overexpressing IFIT1 or IFIT1 in combination with IFIT3 (Fig. S8). WT replication was not affected by any combination of IFIT1 and IFIT3 overexpression, suggesting the ability of WT SARS-CoV-2 to control the antiviral action of these IFIT family members. Together, the results suggest that IFIT1 overexpression is sufficient to attenuate dNSP16, likely due to recognition of nonhost RNA cap structures by IFIT1.

### Targeting the NSP16 active site for antiviral treatment.

**FIG 6:**
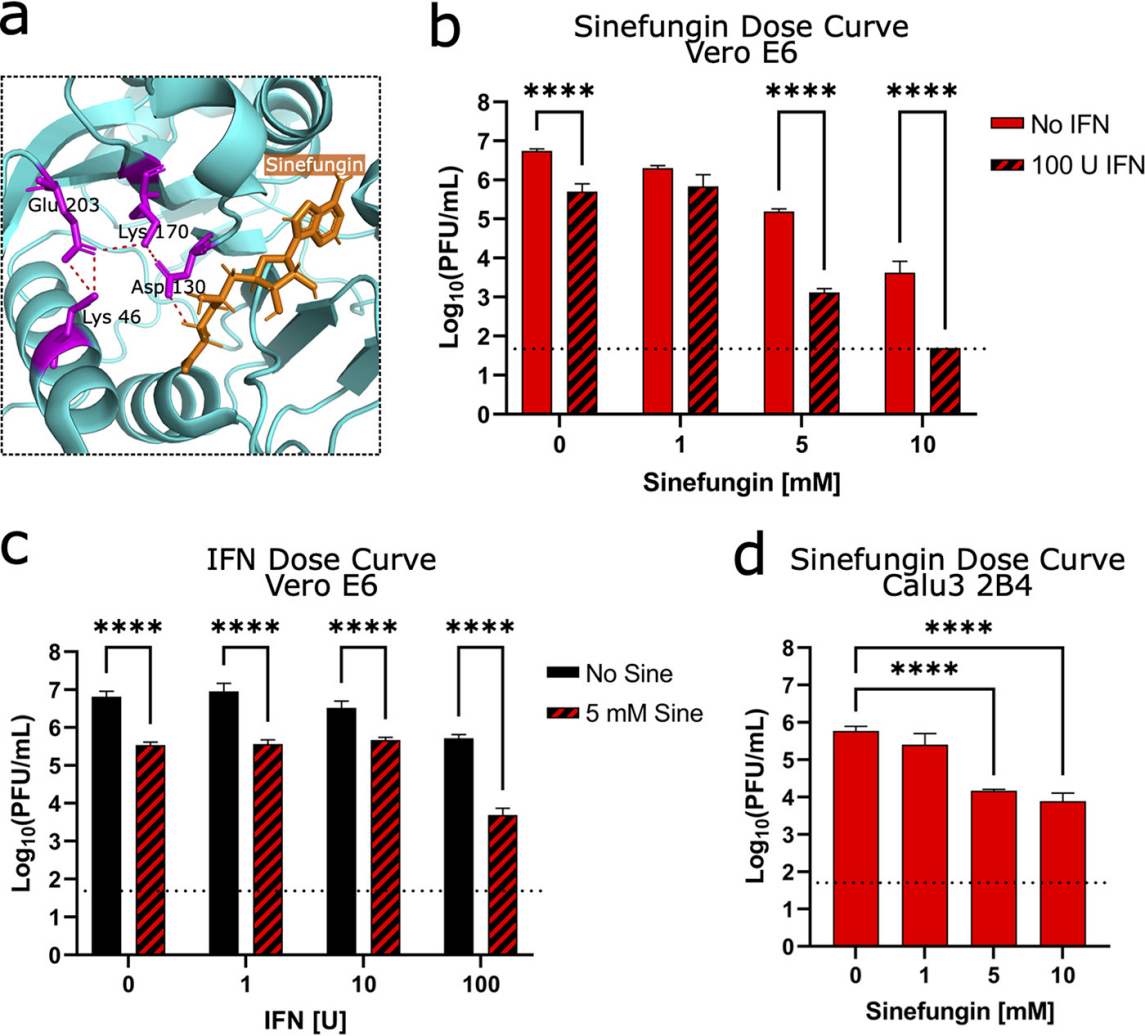
Targeting the NSP16 active site for antiviral treatment. (a) Detail of structure of NSP16 in complex with sinefungin, from Protein Data Bank ID 6YZ1 (36). The residues of the catalytic core are colored in magenta, sinefungin is colored in orange, and polar contacts are shown by orange dashed lines. (b, c) Dose-dependent effect of sinefungin (b) or IFN-I (c) on WT SARS-CoV-2 replication in Vero E6 cells, with or without treatment of the other. A total of 5 × 10^4^ Vero E6 cells/well were seeded in 24-well format 1 day before infection and also pretreated with 100 U IFN-I 8 h later. The day of infection (multiplicity of infection = 0.01), sinefungin was given at the indicated concentration(s) 1 h after infection (in cell culture media). ****, *P < *0.001: results of two-way ANOVA with Tukey’s multiple-comparison test (*α* = 0.05). (d) Dose-dependent effect of sinefungin on WT SARS-CoV-2 replication in Calu3 2B4 cells. Sinefungin was given at the indicated concentrations 1 h after infection (in cell culture media). ****, *P < *0.001: results of one-way ANOVA with Tukey’s multiple-comparison test (*α* = 0.05). For all panels, the data shown are from 48 HPI. The means are plotted with error bars denoting standard deviation (*n *= 3 biological replicates for all data points).

Having established the critical role for NSP16 in helping SARS-CoV-2 evade IFIT function, we next explored whether NSP16 activity could be targeted for therapeutic treatment. Using sinefungin, an *S*-adenosyl-l-methionine (SAM) analog and inhibitor of SAM-dependent MTases (35), we attempted to disrupt NSP16 MTase activity and reduce replication of WT SARS-CoV-2. Previous modeling studies demonstrated that sinefungin binds in the active site of NSP16, interacting with the D130 residue we mutated in dNSP16 (Fig. 6a) (36). We tested a range of sinefungin concentrations on WT SARS-CoV-2 replication in Vero E6 cells. We observed a dose-dependent decrease in WT SARS-CoV-2 replication, with 5 and 10 mM concentrations reducing replication by 1.6 and 3.1 log_10_, respectively (Fig. 6b, solid bars). However, we did note mild and moderate toxicity of sinefungin treatment on cell viability at 5 mM (4%) and at 10 mM (23%), respectively (Fig. S9). The results indicate dose limitations to using sinefungin as a therapeutic treatment but confirm efficacy of targeting MTase activity for reducing viral titer.

Our earlier data suggest that disruption of NSP16 activity will sensitize SARS-CoV-2 to IFN-I-induced effectors like IFIT1 and IFIT3 which could allow the use of a lower dose of sinefungin for treatment. Therefore, we tested the additive impact of sinefungin and IFN-I pretreatment used in combination (Fig. 6b, striped bars). Our results indicated that the combination of sinefungin with a constant dose of IFN-I pretreatment drove further attenuation of WTSARS-CoV-2 to replication levels similar to those observed with dNSP16 (Fig. 2b). Similarly, a constant dose of sinefungin paired with increasing IFN-I amounts also further reduced SARS-CoV-2 replication (Fig. 2c). Overall, sinefungin and IFN-I pretreatment in combination led to a reduction in titer beyond that of either treatment alone, suggesting a possible treatment approach for SARS-CoV-2 and future emergent CoVs.

Finally, we tested sinefungin treatment in Calu3 2B4, which are IFN-I competent and express baseline levels of IFIT proteins (Fig. 5b). Similar to Vero E6 cells, we observed a dose-dependent decrease in SARS-CoV-2 replication, with 5 and 10 mM sinefungin concentrations reducing replication by 1.6 and 1.9 log_10_, respectively (Fig. 6d). Notably, we observe less sinefungin toxicity in Calu3 2B4 cells, indicating cell type differences in susceptibility (Fig. S9). Overall, these results argue that targeting NSP16 MTase function in the context of IFN-I signaling sensitizes SARS-CoV-2 to induced effectors and offers a novel approach for therapeutic CoV treatments.

## DISCUSSION

In this study, we engineered NSP16-mutant SARS-CoV-2 with an amino acid change at a conserved catalytic residue, D130A. The mutant, dNSP16, replicated similarly to WT SARS-CoV-2 in the IFN-I-deficient cell line Vero E6 but was attenuated in human respiratory cells. Moreover, dNSP16 showed greater sensitivity to pretreatment with exogenous IFN-I compared to WT. *In vivo*, dNSP16 was attenuated compared to WT, as evidenced by decreased weight loss, lack of clinical signs of disease, and reduced pathological changes in the hamster lung. Attenuated disease corresponded to lower viral titers in the nasal wash and lung, as well as reduced viral antigen staining in the lung. Mechanistically, the attenuation of dNSP16 is mediated by IFIT1 and IFIT3, with knockdown or overexpression of either gene impacting viral replication of the dNSP16 mutant. Last, we found that sinefungin, an *S*-adenosyl-l-methionine (SAM) analog that targets NSP16 activity, reduced WT SARS-CoV-2 replication. In addition, the effect of sinefungin on reducing viral replication was enhanced when combined with IFN-I pretreatment, likely as a result of decreased NSP16 MTase function and a corresponding increase in recognition by IFIT proteins. Together, our work highlights the critical role of NSP16 in neutralizing the antiviral effects of IFIT1/IFIT3 against WT SARS-CoV-2.

Ablating NSP16 MTase activity does not result in loss of the replicative capacity of dNSP16 compared to WT in IFN-I-incompetent cells. However, our inability to rescue an NSP16-deletion virus with an inserted stop codon suggests that NSP16’s role may be more complex than its 2′-*O*-MTase activity alone. Notably, replication attenuation of dNSP16 occurs in the context of a viable IFN-I response. These results are consistent with previous studies of 2′-*O*-MTase mutants in CoVs, including SARS-CoV (15) and MERS-CoV (23), and recent studies with SARS-CoV-2 (37, 38). Similarly, reduced disease and attenuation of viral replication at 4 DPI in the lungs of dNSP16-infected hamsters is consistent with data from other 2′-*O*-MTase CoV mutants in mouse and hamster models (15, 23, 39). However, our viral titer data from nasal washes, a measure of viral fitness in the upper airway, indicate that dNSP16 attenuation occurs at the earlier 2-DPI time point. These viral titer data from nasal washes, not surveyed in the CoV mouse models, suggest that the upper airway and the lung have distinct immune activation responses, leading to different kinetics for dNSP16 attenuation. One possibility is activation of type III IFN pathways, which is more common at mucosal surfaces (40); both IFIT1 and IFIT3 are among the most highly activated genes following IFNλ stimulation (41). In addition, both IRF3 and IRF7 can induce IFIT expression independent of classic type I IFN signaling (42). As such, further study of the differences between the upper and lower airway are required to understand the earlier attenuation observed from the dNSP16 mutant.

Our studies also confirm a role for the IFIT proteins in mediating attenuation of dNSP16. Previously, human IFIT1, an ISG, has been shown to sequester viral mRNA lacking 2′-*O*-methylation (43) through a mechanism that involves direct recognition of the cap structure (44). In prior studies with CoVs, mouse Ifit1, paralogous to human IFIT1 (45), antagonized CoVs lacking 2′-*O*-methylation (10, 11). Here, we demonstrate that while dNSP16 is attenuated by IFN-I pretreatment in Vero E6 cells, knockdown of IFIT1 partially restores dNSP16 replication. In addition, rapid attenuation of dNSP16 in Calu-3 2B4 cells, compared to Vero E6 cells, may be due to higher baseline levels of IFIT1 in the former. We also found that knockdown of IFIT3 partially restored dNSP16 replication in the context of IFN-I pretreatment. Recent studies have highlighted the importance of IFIT3 in stabilizing IFIT1 function and optimizing its recognition of RNA caps lacking 2′-*O*-methylation (33). Notably, the combination of IFIT1 and IFIT3 knockdown that we tested had no additive effect, suggesting that both together are required for restriction of dNSP16. Conversely, we found that overexpression of IFIT1 was sufficient for modest attenuation dNSP16 with no impact on WT SARS-CoV-2. Overall, these results indicate the importance of NSP16 in protecting CoVs from IFIT effector function.

Having established a critical role for NSP16 in evading IFIT activity, we evaluated the feasibility of targeting 2′-*O*-methylation of CoVs therapeutically. Using sinefungin, a pan-inhibitor of SAM-dependent MTases, we surprisingly observed a dose-dependent reduction in replication of WT SARS-CoV-2 in Vero E6. Since we had not observed a replication defect with dNSP16 in Vero E6 (Fig. 1c), it is possible that sinefungin acts beyond NSP16 alone. A pan-MTase inhibitor, sinefungin may also impact the guanine-N7 MTase function of NSP14, which is also vital for viral RNA capping (37). Alternatively, sinefungin may also act on a host cell MTase(s), which have been recently implicated in SARS-CoV-2 RNA capping (38). While further studies are necessary to differentiate this effect, our results indicate that targeting SAM-dependent MTases impairs successful SARS-CoV-2 replication.

Importantly, combined treatment with sinefungin and IFN-I had an additive effect, resulting in increased attenuation likely due to both a loss of viral 2′-*O*-methylation and increased expression of IFIT1/IFIT3. These results correspond to a similar study with SAM cycle inhibitors that saw the same effect (37). While IFN-I treatments (both IFNα and IFNβ) have shown significant impacts in randomized clinical trials (46), our results suggest that combining such treatments with an NSP16-targeting drug could enhance their impact by reducing toxicity by lowering the therapeutic dose. Notably, targeting NSP16 MTase function involves a mechanism distinct from other CoV therapies targeting the viral polymerase (47) or the main protease (48) to arrest virus replication. In each case, a viral enzymatic process is disrupted; yet for NSP16 targeting, the effector response is provided by the host immune response via IFIT proteins. Importantly, while attenuation of dNSP16 is delayed in the hamster lung, early attenuation in the nasal washes suggests more rapid or robust expression of IFIT proteins in the upper airway. This could, in turn, increase the efficacy of drugs targeting CoV 2′-*O*-MTase activity in the upper airway, a possible strategy to decrease transmission. With augmented upper airway replication a feature of SARS-CoV-2 variants of concern (49), NSP16-targeting drugs may provide an effective countermeasure for the current and future CoV.

Overall, our results confirm the importance of NSP16 to SARS-CoV-2 infection and pathogenesis. A mutation that disrupts the NSP16 2′-*O*-MTase catalytic site attenuates disease *in vivo* and demonstrates its importance in evading host innate immunity. In the absence of 2′-*O*-MTase activity, SARS-CoV-2 is rendered susceptible to the effector responses of IFIT1 and IFIT3 in combination. Importantly, such dependence of SARS-CoV-2 on the 2′-*O*-MTase function of NSP16 offers a novel target for future CoV antiviral drug development.

## MATERIALS AND METHODS

### Cells.

Vero E6 cells (ATCC No. CRL-1586) were cultured in high-glucose Dulbecco’s modified Eagle’s medium (DMEM, Gibco No. 11965-092) supplemented with 5% heat-inactivated fetal bovine serum (FBS, Cytiva No. SH30071.03) and 1× antibiotic-antimycotic (Gibco No. 15240-062). VeroE6/TMPRSS2 (JCRB No. 1819) were cultured in low-glucose, pyruvate-containing DMEM (Gibco No. 11885-084) supplemented with 5% FBS and 1 mg/mL Geneticin (Gibco No. 10131-035). Calu-3 2B4 (BEI Resources No. NR-55340) was cultivated in high-glucose DMEM supplemented with 10% FBS, 1× antibiotic-antimycotic, and 1 mM sodium pyruvate (Sigma-Aldrich No. S8636). Baby hamster kidney (BHK) cells were cultured in α-minimal essential medium (α-MEM) with GlutaMAX (Gibco No. 32561-037) supplemented with 5% FBS and 1× antibiotic-antimycotic. For all propagation and experimentation, the cells were kept at 37°C and 5% CO_2_ in a humidified incubator.

### Viruses.

We performed PCR-based mutagenesis to engineer a 2-bp mutation in codon 130 of the NSP16 gene encoded on a SARS-CoV-2 infectious clone (ic) reverse genetics system based on the prototype USA/WA1/2020 strain (NCBI accession No. MN985325), following our previously published method (19, 20). The engineered change was made to the second and third bp positions of NSP16 codon 130 (*GAT*→*GCG*) on pUC57-CoV-2-F5, changing the encoded aspartic acid residue to an alanine. The initially rescued virus constituted a heterogenous population of sequences; therefore, the initial stock was serially diluted and plated into wells containing Vero E6 cells to isolate single clones via plaque purification. Individual plaques were carefully scraped with a pipette tip and used to inoculate separate wells containing Vero E6 cells. Upon induction of CPE, culture supernatants were cleared of cellular debris, and part of the liquid fraction was processed for viral RNA purification and Sanger sequencing. Well supernatants associated with viral sequences that contained the desired NSP16 mutation were then used to infect TMPRSS2-expressing Vero E6 cells for an additional round of virus replication to generate higher viral titers; TMPRSS2-expressing cells were chosen to reduce the chance of mutation of the spike protein around the furin cleavage site (24). The supernatants from these cells were similarly processed as described above for confirmation of viral sequence via Sanger sequencing. Upon sequence verification, a supernatant stock of icSARS-CoV-2 with the engineered NSP16 mutation (“dNSP16”) was selected for use in subsequent experiments. With the exception of the plaque purification step, wild-type icSARS-CoV-2 (WT) was produced in the same way as dNSP16.

### Viral replication kinetics.

Cells were seeded in 24-well format. In experiments involving IFN-I pretreatment, the cells were treated for 16 to 20 h prior to infection with Universal Type I IFN (PBL Assay Science No. 11200-2), diluted in Dulbecco’s phosphate-buffered saline, without calcium chloride and magnesium (DPBS, Gibco No. 14190-144). After infection at a multiplicity of infection (MOI) of 0.01 and incubation for 45 min at 37°C with 5% CO_2_ and manual tilting every 15 min, the cells were washed three times with 500 μL DPBS and then given 500 μL of cell type-specific medium. Supernatants were collected within 1 h of the indicated time point, whereupon 150 μL of culture medium was removed, and an equal volume of fresh medium was added back to the sample well. The titers of the supernatant samples were subsequently determined via plaque assay. All conditions were performed in triplicate, and all experiments were performed in an approved biosafety level 3 (BSL3) laboratory at the University of Texas Medical Branch at Galveston (UTMB).

### Plaque assay.

One day before the assay, 6-well plates were seeded with 3 × 10^5^ Vero E6 cells/well. Under BSL3 conditions, samples of virus-containing supernatant were titrated in a 10-fold dilution series in DPBS, and 200 μL of each dilution of the series was transferred to confluent cells after the culture medium was removed. The assay plates were incubated at 37°C with 5% CO_2_ for 45 min with manual tilting every 15 min. Afterwards, an overlay of 1× modified Eagle’s medium (Gibco No. 11935-046) containing 5% heat-inactivated FetalClone II (Cytiva No. SH30066.03), 1× antibiotic-antimycotic, and 1% agarose (Lonza No. 50004) was applied to wells, and the plates were returned to the incubator for 2 days. Afterwards, a 1× dilution in DPBS of 10× neutral red stain (0.85% wt/vol NaCl, 0.5% wt/vol Fisher Scientific No. N129-25) was applied to each well, and 2 to 5 h later, PFU were visualized using a lightbox and manually counted. The limit of detection was 50 PFU/mL, corresponding to 1 PFU in the well with the lowest dilution factor (1:50 total dilution).

### Animal studies.

Four- to five-week-old male Syrian hamsters (Mesocricetus auratus), strain HsdHan:AURA, purchased from Envigo were infected intranasally (i.n.) with a 10^4^ PFU dose of either dNSP16 or WT in a 100-μL inoculum volume or DPBS for mock-infected animals. The hamsters were randomly assigned to different treatment groups. Animal weights and clinical signs were recorded daily for up to 7 days postinfection (DPI). The disease scores were as follows: 1 (healthy), 2 (ruffled fur), 3 (hunched posture, orbital tightening, lethargy), and 4 (moribund). At 2, 4, and 7 DPI, nasal washes from five animals from each experimental group were collected, and the animals were subsequently sacrificed, with right cranial, right middle, and left lung lobes from each animal collected in either DPBS, RNAlater (Invitrogen No. AM7021), or 10% phosphate-buffered formalin (Fisher No. SF100) for subsequent analyses of viral titer, gene expression and viral sequence, or histopathology, respectively. For measurement of viral titer, collected lung lobes were homogenized at 6,000 rpm for 60 s using a Roche MagNA Lyser instrument, and then their titers were determined via plaque assay. For analysis of gene expression and viral sequence, lung lobes stored in RNAlater were transferred to TRIzol (Invitrogen No. 15596018) and homogenized five times at 6,500 rpm for 30 s, with cooling on a –20°C-chilled rack for 1 min between homogenization steps. The homogenates were then processed for RNA purification as described below. For histopathological analysis, lung lobes were incubated with 10% phosphate-buffered formalin for 7 days at 4°C to allow for deactivation and buffer exchange before processing. All animal handling was performed at animal biosafety level 3 (ABSL3) conditions and in accordance with guidelines set by the Institutional Animal Care and Use Committee (IACUC) of the University of Texas Medical Branch.

### Histology.

For visualization of histopathology, sections of paraffin-embedded formalin-fixed tissue were stained with hematoxylin and eosin on a SAKURA VIP 6 tissue processor at the University of Texas Medical Branch Surgical Pathology Laboratory. For visualization of viral antigen, tissue sections were deparaffinized and stained with a SARS-CoV-2 N-specific rabbit monoclonal antibody (Sino Biological No. 40143-R001) at a dilution of 1:30,000 followed by an anti-rabbit horseradish peroxidase (HRP)-linked secondary (Cell Signaling No. 7074). A signal was developed with an ImmPact NovaRED peroxidase kit (Vector Labs No. SK-4805). Viral antigen staining was scored blinded on a scale of 0 (none) to 3 (most) in 0.25 score increments.

### RNA purification.

RNA from cell supernatants, cell lysates, or homogenized lung tissue was extracted in TRIzol LS (Invitrogen No. 10296010) for cell supernatants only or TRIzol, followed by purification using Direct-zol RNA Miniprep Plus (Zymo Research No. R2072) and reverse transcription using iScript cDNA synthesis kit (Bio-Rad No. 1708891).

### Sanger sequencing.

Phusion High-Fidelity PCR Master Mix with HF Buffer (New England BioLabs No. M0530) was used to amplify cDNA around the region of interest. A total of 45 amplification cycles were used; otherwise, the manufacturer’s protocol was followed. To amplify the region encoding NSP16, forward primer 5′-AACAGATGCGCAAACAGG and reverse primer 5′-TGCAGGGGGTAATTGAGTTC were used. To amplify the region of spike in the vicinity of the furin cleavage site, forward primer 5′-AGGCACAGGTGTTCTTAC and reverse primer 5′-TGAAGGCTTTGAAGTCTGCC were used. Amplicons were verified by gel electrophoresis, purified using QIAquick PCR purification kit (Qiagen No. 28106), and sent to Genewiz (South Plainfield, NJ) for Sanger sequencing.

### Gene expression via quantitative PCR (qPCR).

qPCR was performed on cDNA using Luna (New England BioLabs No. M3003) according to the manufacturer’s instructions. Fluorescent readings were made on a Bio-Rad CFX Connect instrument using Bio-Rad CFX Maestro 1.1 software (version 4.1.2433.1219). The relative gene expression was calculated manually using the ΔΔCt method: For each cDNA sample, the threshold cycle (Ct) of the gene of interest was first normalized against the Ct of the indicated reference gene. Then, the fold change in normalized expression for the gene of interest in each sample was calculated relative to normalized expression of the gene of interest in the control sample. The primers used for amplifying hamster targets were 18S (forward: 5′-GTAACCCGTTGAACCCCATT; reverse: 5′-GTAACCCGTTGAACCCCATT), pan-IFIT1 (predicted to amplify NCBI accession Nos. XM_021224958, XM_040745240, and XM_013110344, forward: 5′-TGCAGAGCTTGAAAGAAGCA; reverse: 5′-CCTTCCTCACAGTCCACCTC), IFIT3 (forward: 5′-CCTGGAGTGCTTAAGGCAAG; reverse: 5′-TGCCTCACCTTGTCCACATA), RNase L (forward: 5′-CCAGAGGGTAAAAACGTGGA; reverse: 5′-TGCACCAAACCTGTGTGTTT), PKR (forward: 5′-AAGTGCGTGAAGTAAAGGCG; reverse: 5′-ATCCATTGCTCCAGAGTCCC), Mx1 (forward: 5′-CTTCAAGGAGCACCCACACT; reverse: 5′-CTTGCCCTCTGGTGACTCTC), IFNγ (forward: 5′-GGCCATCCAGAGGAGCATAG; reverse: 5′-TTTCTCCATGCTGCTGTTGAA), IL-1β (forward: 5′-GGCTGATGCTCCCATTCG; reverse: 5′-CACGAGGCATTTCTGTTGTTCA), and IL-10 (forward: 5′-GTTGCCAAACCTTATCAGAAATGA; reverse: 5′-TTCTGGCCCGTGGTTCTCT). The primers used for amplifying targets in Vero E6 cells were β-actin (forward: 5′-GGCATCCTCACCCTGAAGTA, reverse: 5′-GGGGTGTTGAAGGTCTCAAA), IFIT1 (forward: 5′-ACACCTGAAAGGCCAGAATG; reverse: 5′-GCTTCTTGCAAATGTTCTCC), IFIT3 (forward: 5′-AGGAAGGGTGGACACAACTG; reverse: 5′-TGGCCTGTTTCAAAACATCA), OAS1 (forward: 5′-GATCTCAGAAATACCCCAGCCA; reverse: 5′-AGCTACCTCGGAAGCACCTT), and PKR (forward: 5′-ACGCTTTGGGGCTAATTCTT; reverse: 5′-TTCTCTGGGCTTTTCTTCCA). All primers were purchased as single-stranded DNA oligomers purified with standard desalting (Integrated DNA Technologies, Coralville, IA.).

### DsiRNA experiments.

The following dicer-substrate short interfering RNAs (DsiRNAs) (Integrated DNA Technologies) were utilized: anti-IFIT1 (sense: 5′-rGrCrUrUrGrArGrCrCrUrCrCrUrUrGrGrGrUrUrCrGrUrCTA; antisense: 5′-rUrArGrArCrGrArArCrCrCrArArGrGrArGrGrCrUrCrArArGrCrUrU), anti-IFIT3 (sense: 5′-rArGrCrUrGrArGrUrCrCrUrGrArUrArArCrCrArArUrArCGT; antisense: 5′-rArCrGrUrArUrUrGrGrUrUrArUrCrArGrGrArCrUrCrArGrCrUrCrA), anti-OAS1 (sense: 5′-rCrGrGrUrCrUrUrGrGrArArUrUrArGrUrCrArUrArArArCTA; antisense: 5′-rUrArGrUrUrUrArUrGrArCrUrArArUrUrCrCrArArGrArCrCrGrUrC), and anti-PKR (sense: 5′-rGrUrArUrUrGrGrUrArCrArGrGrUrUrCrUrArCrUrArArACA; antisense: 5′-rUrGrUrUrUrArGrUrArGrArArCrCrUrGrUrArCrCrArArUrArCrUrA), and negative-control DsiRNA (Integrated DNA Technologies No. 51-01-14-03). For DsiRNA experiments, 1.25× 10^5^ Vero E6 cells/well were reverse transfected in 24-well plate format with 1 to 2 pmol/well DsiRNA as indicated, 2 days prior to infection. At 16 to 20 h prior to infection, cells were treated with 100 U of DPBS-diluted Universal Type I IFN (PBL Assay Science No. 11200-2). Infections proceeded as described in the section “Viral replication kinetics” above.

### Protein expression via Western blot.

The cell lysates were harvested with 2× Laemmli SDS-PAGE sample buffer (Bio-Rad No. 1610737) containing a final concentration of 5% β-mercaptoethanol (Bio-Rad No. 1610710). The cell lysates were then denatured at 95°C for 10 min. The lysates were then loaded onto a Mini-PROTEAN TGX gel (Bio-Rad No. 4561096) and electrophoresed, followed by transfer to a polyvinylidene difluoride membrane (Bio-Rad No. 1620177). The membrane was then blocked in 5% nonfat dry milk dissolved in Tris-buffered saline with 0.1% Tween 20 (TBS-T) for 1 h, followed by a short TBS-T wash. Overnight incubation with primary antibody, either rabbit anti-hIFIT1 (Cell Signaling Technology No. 14769) or rabbit anti-β-actin (Cell Signaling Technology No. 4970) was then performed. Afterward, the membrane was washed three times with TBS-T and incubated with horseradish peroxidase-conjugated secondary antibody (Cell Signaling Technology No. 7074) for 1 h. Finally, the membrane was washed three times with TBS-T, incubated with Clarity Western ECL Substrate (Bio-Rad No. 1705060) and imaged with a Bio-Rad ChemiDoc Imaging System running Bio-Rad Image Lab Touch software (version 2.4.0.03).

### Overexpression experiment.

A total of 8.5× 10^4^ Huh7 cells/well were reverse transfected with 0.8 μg total of pcDNA3-EGFP (a gift from Doug Golenbock, Addgene No. 13031), pcDNA3.1 3×Flag IFIT1 (50) (a gift from Kathleen Collins, Addgene No. 53554), pcDNA3.1 3×Flag IFIT3 (50) (a gift from Kathleen Collins, Addgene No. 53553), or both pcDNA3.1 3×Flag IFIT1 and pcDNA3.1 3×Flag IFIT3 2 days prior to infection. pcDNA3 and pcDNA3.1 have minor differences in restriction sites but the same cytomegalovirus (CMV) immediate early promoter. Infections proceeded as described in the section “Viral replication kinetics” above.

### Viability assay.

A total of 1.0× 10^3^ cells/well were seeded in 96-well format. One day later, the cells were treated with sinefungin, in parallel to infection experiments with sinefungin. Two days after sinefungin treatment, cell viability was measured using CellTiter-Glo luminescent cell viability assay (Promega No. G7570). Luminescence was read on a Tecan Infinite 200 PRO running Tecan i-control software (version 2.0.10.0), using an integration time of 1 s.

### Statistics.

All statistics were performed in GraphPad Prism 9 (version 9.0.2), with details given in the figure legends. In summary, two-way analysis of variance (ANOVA) was performed on log_10_-transformed viral titers, with Tukey’s multiple-comparison test (*α* = 0.05) to infer significant differences. For gene expression or cell viability data, one-way ANOVA was performed on ΔΔCt values or relative luminescence values, respectively, with Tukey’s multiple-comparison test (*α* = 0.05) to infer significant differences. For comparing viral load via qPCR, a one-tailed *t* test was performed. For scoring of antigen presence in histology samples, two-way ANOVA was performed on scores, with Šídák's multiple-comparison test (*α* = 0.05) to infer significant differences. For animal weight data, a mixed-effects model (restricted maximum likelihood) was used, with Tukey’s multiple-comparison test (*α* = 0.05) to infer significant differences. For animal experiments, a group size of *n *= 5 animals per condition per time point was chosen based on previous studies (27). For all data at or below the limit of detection, values were set to the limit of detection.
