# Supplementary material for: SARS-CoV-2 Uses Nonstructural Protein 16 To Evade Restriction by IFIT1 and IFIT3
Source: J Virol. 2023 Feb 1;97(2):e01532-22. doi: 10.1128/jvi.01532-22 (PMC9973020; doi:10.1128/jvi.01532-22)
Supplement: Supplemental file 1 — Fig. S1 to S9. Download jvi.01532-22-s0001.pdf, PDF file, 6.0 MB [file jvi.01532-22-s0001.pdf]

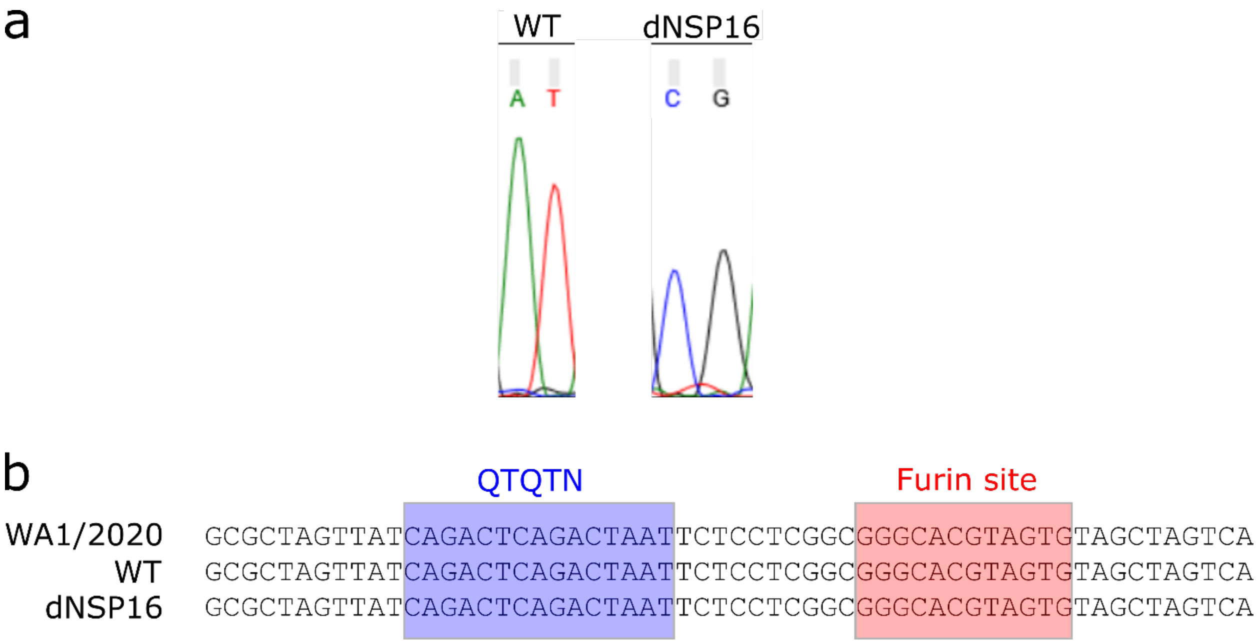

Figure S1. D130 mutation is stable in rescued dNSP16, and rescued infectious clone stocks maintain sequence around furin cleavage site. Viral RNA was extracted from the viral stocks used in the study ("WT" and "dNSP16"). Viral RNA was reverse-transcribed, PCR-amplified around the site of interest, and Sanger sequenced. (a) Shown are the sequencing traces of the 2-base pair site within codon 130 of NSP16 that was mutated from AT to CG to engineer dNSP16. (b) Validated sequence around the furin cleavage site, including the QTQTN motif, for WT and dNSP16, compared to the published sequence for WA1/2020.

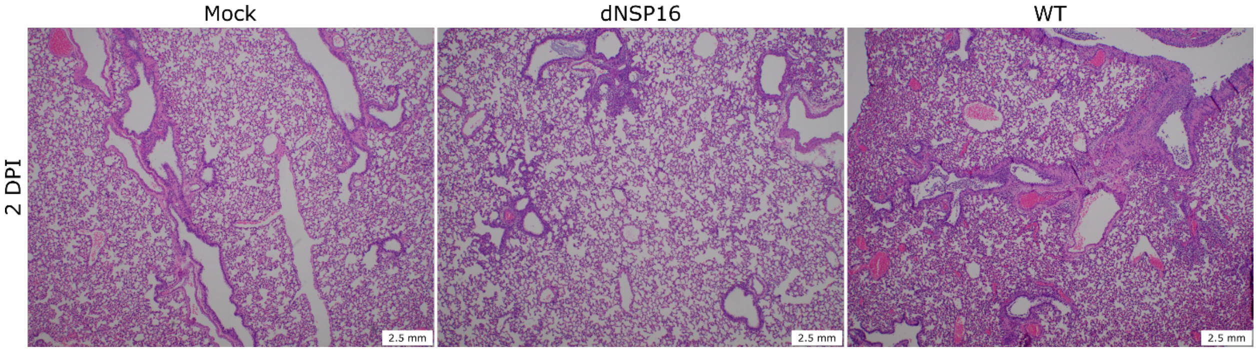

Figure S2. dNSP16 is attenuated *in vivo*. A 100 µL inoculum of PBS (mock) or 10^4 plaque-forming units of either dNSP16 or WT was given intranasally to 4- to 5-week-old Syrian hamsters. Shown are hematoxylin and eosin staining of representative 5 µm-thick sections taken from left lung lobes of animals sacrificed at 2 days post-infection (DPI).

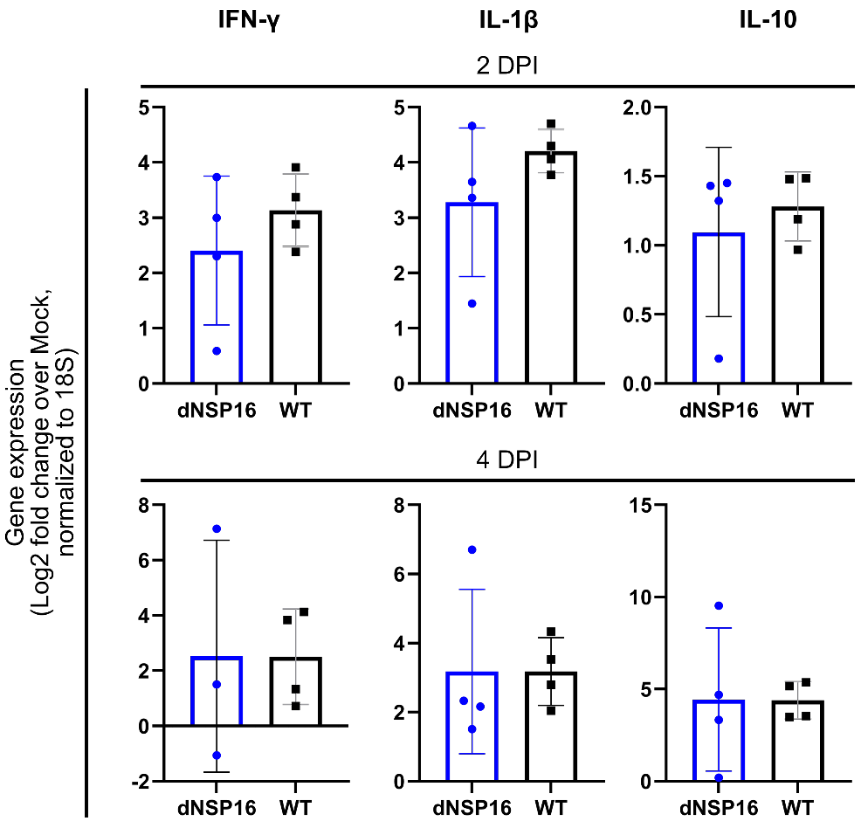

Figure S3. dNSP16 does not drive increased immune gene expression relative to WT. Fold change ($\log_2$) of expression of the indicated immune genes from lung samples isolated from hamsters infected with the indicated virus, 2 or 4 days post-infection. For each panel, fold changes from dNSP16 or WT samples are measured relative to mock samples. Values from individual hamsters are plotted (symbols) as well as means (bars). Error bars denote standard deviation. All samples were normalized to *18S* expression, used as a reference.

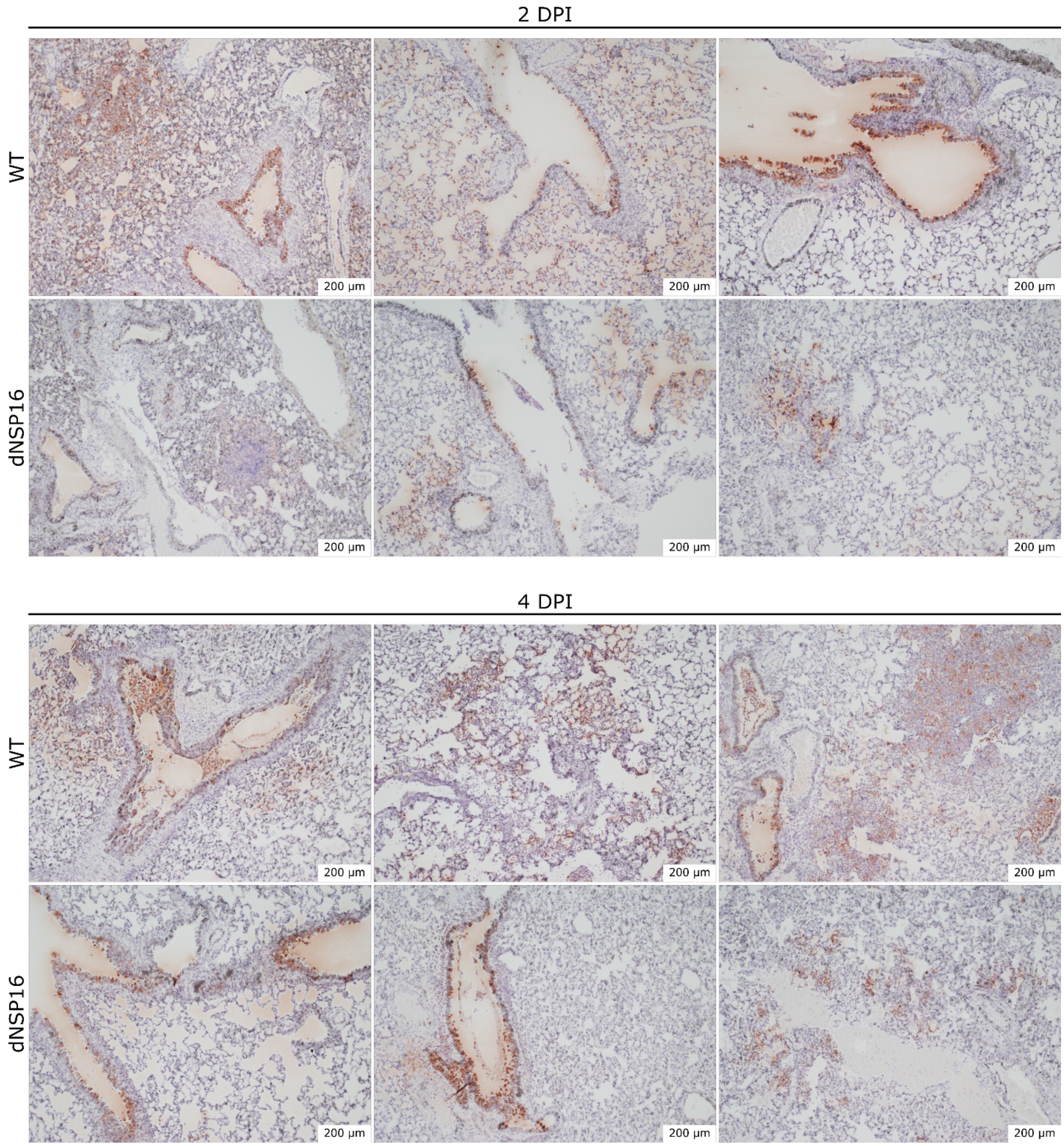

Figure S4. dNSP16 replication is reduced *in vivo*. SARS-CoV-2 nucleocapsid staining (brown) of representative 5 µm-thick sections taken from left lung lobes of mock-, dNSP16, or WT-infected hamsters at the indicated day post-infection (DPI).

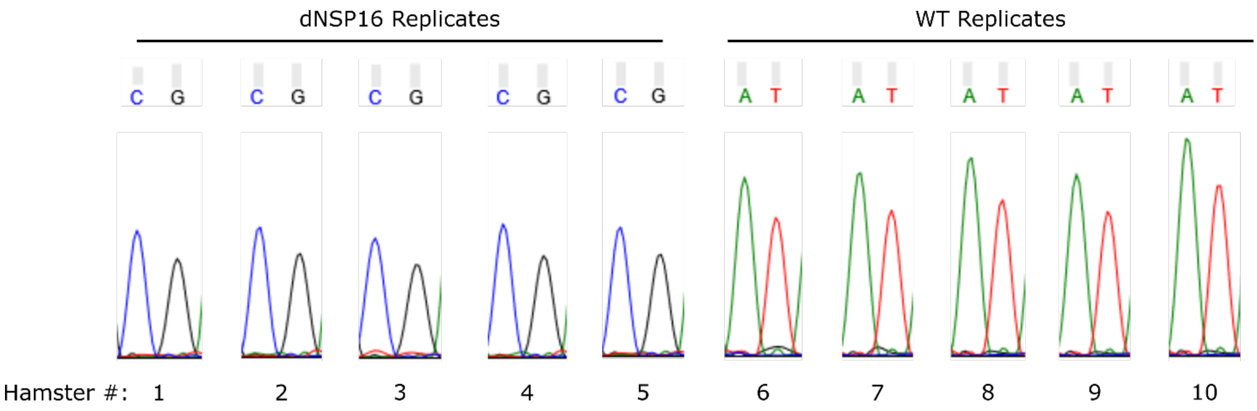

Figure S5. No evidence of reversion of dNSP16 mutation was detected *in vivo*. Viral RNA was extracted from the lungs of hamsters infected with either dNSP16 or WT (numbered 1 through 5 for each group) and which were sacrificed at 4 days post-infection. Viral RNA was reverse-transcribed, PCR-amplified around the site of mutation, and Sanger sequenced. Shown are the sequencing traces of the 2-base pair site within codon 130 of NSP16 that was mutated from AT to CG to engineer dNSP16.

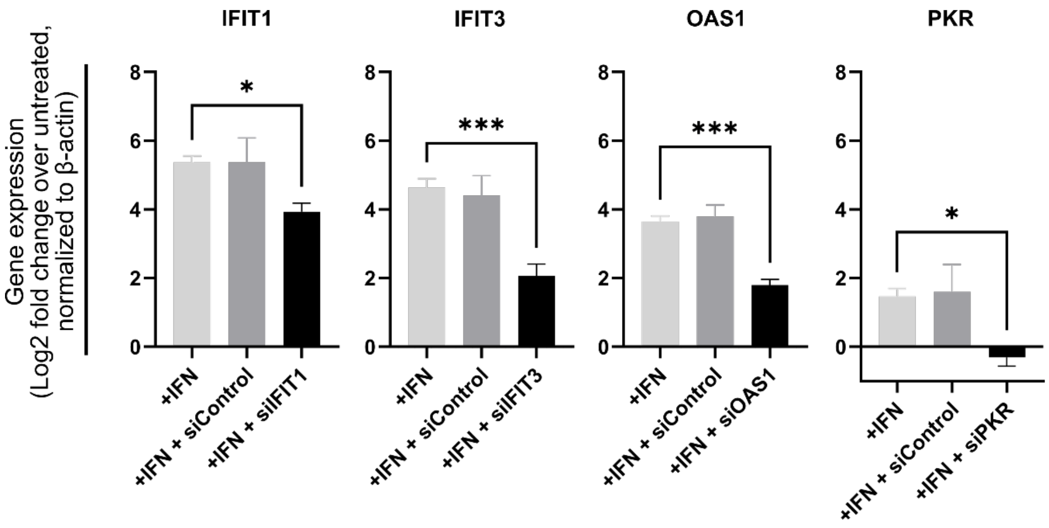

Figure S6. Validation of knockdown of immune gene targets in Vero E6 cells. 1.25 x 10^5 Vero E6 cells/well were reverse transfected with 1 pmol of the control or gene-specific siRNA 2 days prior to harvest and also treated with 100 U IFN-I one day prior to harvest and assessment of gene expression. Fold change (log_2) of gene expression is measured relative to untreated samples (i.e. no IFN-I). All samples were normalized to *β*-actin, used as a reference. *$p<0.05$, ***$p<0.005$, ns = not significant: results of one-way ANOVA with Tukey's multiple comparison test ($\alpha = 0.05$). Means are plotted with error bars denoting standard deviation. $n = 3$ biological replicates.

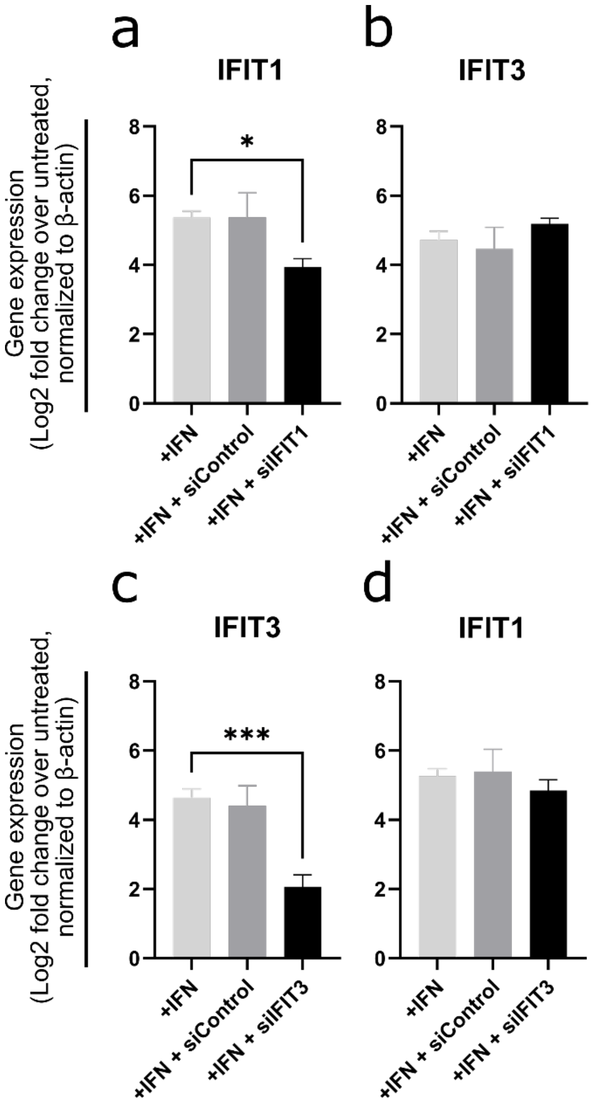

Figure S7. Knockdown of either *IFIT1* or *IFIT3* is specific. 1.25×10^5 Vero E6 cells/well were reverse transfected with 1 pmol/well of either a non-targeting siRNA ("siControl") or with an *IFIT1*- (a, b) or *IFIT3*- (c, d) targeting siRNA ("siIFIT1" or "siIFIT3", respectively), or were seeded without treatment. One day later, cells were treated with 100 U of IFN-I to induce interferon-stimulated genes. The following day, cells were lysed for RNA purification and mRNA quantification via reverse transcription and quantitative polymerase chain reaction (PCR). For all panels, gene expression is normalized to *β*-actin (used as a reference), and fold changes are given relative to untreated controls (i.e. no IFN). *$p<0.05$, ***$p<0.005$, ns = not significant: results of one-way ANOVA with Tukey's multiple comparison test ($\alpha = 0.05$). Means are plotted with error bars denoting standard deviation. $n = 3$ biological replicates.

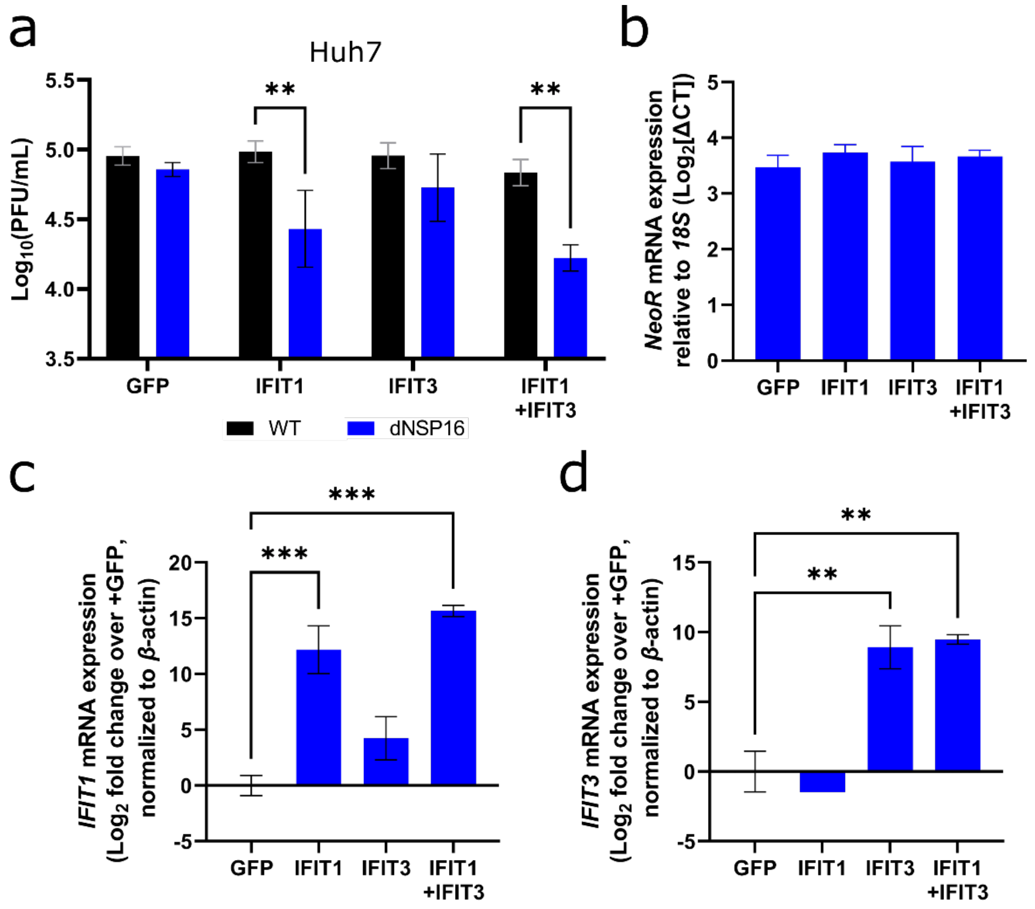

Figure S8. Overexpression of *IFIT1* results in attenuation of dNSP16. (a) Replication of WT (black) and dNSP16 (blue) in the context of overexpression of IFIT plasmids. 8.5 x 10^4 Huh7 cells/well were reverse transfected with 0.8 µg total of plasmid(s) expressing the indicated protein(s) 2 days prior to infection, multiplicity of infection (MOI) = 0.01. Data shown at 50 hours post-infection (HPI). $^{**}p<0.01$: results of two-way ANOVA with Tukey's multiple comparison test (α = 0.05), n = 3. (b – d) Relative quantification of (b) *NeoR* (plasmid backbone), (c) *IFIT1*, or (d) *IFIT3* expression from RNA isolated from unclarified supernatants at 25 HPI from the same dNSP16-infected samples as in (a). $^{**}p<0.01$, $^{***}p<0.005$: results of one-way ANOVA with Tukey's multiple comparison test (α = 0.05). For all panels, means are plotted with error bars denoting standard deviation. PFU = plaque-forming units.

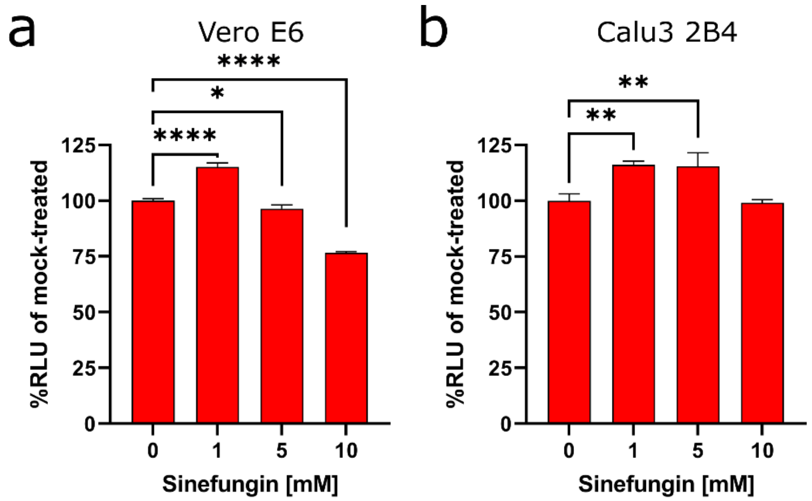

Figure S9. Sinefungin decreases Vero E6, but not Calu3 2B4 viability. (a, b) Cell viability post-sinefungin treatment as measured by amount of luminescence detected in an ATP detection assay. (a) 1 x 10^3 Vero E6 cells/well or (b) 1.5 x 10^3 Calu3 2B4 cells/well were seeded in 96-well format one day before sinefungin treatment. Data shown at 48 hours post-treatment. *$p<0.05$, **$p<0.01$, ****$p<0.001$: results of one-way ANOVA with Tukey's multiple comparison test ($\alpha = 0.05$). For all panels, means are plotted with error bars denoting standard deviation. n = 3 biological replicates for all data points. RLU = relative light units. PFU = plaque-forming units.
